# Strengthening Mental Abilities with Relational Training (SMART) in multiple sclerosis (MS): study protocol for a feasibility randomised controlled trial

**DOI:** 10.1186/s40814-022-01152-7

**Published:** 2022-09-03

**Authors:** Nima Golijani-Moghaddam, David L. Dawson, Nikos Evangelou, James Turton, Annie Hawton, Graham R. Law, Bryan Roche, Elise Rowan, Rupert Burge, Alexandra C. Frost, Roshan das Nair

**Affiliations:** 1 School of Psychology, University of Lincoln, Sarah Swift Building, Brayford Pool, Lincoln, LN6 7TS UK; 2 School of Medicine, University of Nottingham, C floor, South Block, Queen’s Medical Centre, Nottingham, NG7 2UH UK; 3 Institute of Mental Health, Innovation Park, Triumph Road, Nottingham, NG7 2TU UK; 4 University of Exeter, Stocker Rd, Exeter, EX4 4PY UK; 5 Lincoln Clinical Trials Unit (LinCTU), Community and Health Research Unit, School of Health and Social Care, University of Lincoln, Brayford Pool, Lincoln, LN6 7TS UK; 6 Department Psychology, Maynooth University, Co. Kildare, Ireland; 7 Nottinghamshire Healthcare NHS Foundation Trust, Nottingham, UK

**Keywords:** Multiple sclerosis, Cognitive rehabilitation, Feasibility randomised controlled trial, Relational training

## Abstract

**Background:**

Multiple sclerosis (MS) is a chronic condition of the central nervous system, affecting around 1 in every 600 people in the UK, with 130 new diagnoses every week. Cognitive difficulties are common amongst people with MS, with up to 70% experiencing deficits in higher-level brain functions—such as planning and problem-solving, attention, and memory. Cognitive deficits make it difficult for people with MS to complete everyday tasks and limit their abilities to work, socialise, and live independently. There is a clear need—and recognised research priority—for treatments that can improve cognitive functioning in people with MS. The absence of effective cognitive interventions exacerbates burdens on the services accessed by people with MS—requiring these services to manage sequelae of untreated cognitive deficits, including reduced quality of life, greater disability and dependence, and poorer adherence to disease-modifying treatments. Our planned research will fill the evidence gap through developing—and examining the feasibility of trialling—a novel online cognitive rehabilitation programme for people with MS (SMART). The SMART programme directly trains relational skills (the ability to flexibly relate concepts to one another) based on theory that these skills are critical to broader cognitive functioning.

**Methods:**

The primary objective of this study aims to conduct a feasibility study to inform the development of a definitive trial of SMART for improving cognitive functioning in people with MS. The secondary objective is to develop the framework for a cost-effectiveness analysis alongside a definitive trial, and the exploratory objective is to assess the signal of efficacy.

**Discussion:**

As a feasibility trial, outcomes are unlikely to immediately effect changes to NHS practice. However, this is a necessary step towards developing a definitive trial—and will give us a signal of efficacy, a prerequisite for progression to a definitive trial. If found to be clinically and cost-effective, the latter trial could create a step-change in MS cognitive rehabilitation—improving service delivery and optimising support with limited additional resources.

**Trial registration:**

Registration ID: ClnicalTrials.gov: NCT04975685—registered on July 23rd, 2021.

Protocol version: 2.0, 25 November 2021

**Supplementary Information:**

The online version contains supplementary material available at 10.1186/s40814-022-01152-7.

## Background

Multiple sclerosis (MS) is a chronic condition of the central nervous system, affecting around 1 in every 600 people in the UK, with 130 new diagnoses every week [1]. Common symptoms of MS include limb weakness, fatigue, and pain. These symptoms typically come in waves (as ‘attacks’)—lasting weeks before remitting—but often, over time, become permanent, leading to increased disability and physical decline.

Cognitive difficulties are common amongst people with MS, with up to 70% experiencing deficits in higher-level brain functions [2]—such as planning and problem-solving, attention, and memory. In a national survey, these cognitive difficulties were identified as the most debilitating and distressing consequence of MS [3]. Cognitive deficits make it difficult for people with MS to complete everyday tasks and limit their abilities to work, socialise, and live independently [4]—abilities integral to wellbeing [5]. Natural history studies of cognitive dysfunction in MS indicate that deficits are unlikely to improve and often worsen [6]—with great costs to people with MS, their families, and society [7].

There is a clear need—and recognised research priority—for treatments that can improve cognitive functioning in people with MS [8]. Whilst there has been progress in diagnosing cognitive difficulties, efficacious treatment options remain elusive [9, 10]. However, one of the more promising treatment pipelines is cognitive rehabilitation [11]—a structured set of activities to retrain cognitive skills or to improve coping with cognitive deficits in daily life. Whilst several reviews have found some positive effects of cognitive rehabilitation in people with MS, these are based on poor quality randomised controlled trials (RCTs) [12, 13]. More recent, robustly-designed studies, however, are encouraging [9, 14]and suggest that cognitive retraining can be effective for focal deficits (e.g. intensive attentional training for attentional deficits), but questions remain as to the breadth, practical importance, reproducibility, and real-world scalability of such interventions.

Studies to date have typically not been predicated on a clear theoretical rationale for intervention, nor sought to examine possible mechanisms of change. This is problematic as, in the absence of a theoretical framework or process-based examination, it is difficult to synthesise across studies and understand or optimise intervention effects. Currently, no evidence-based recommendations exist for either practice standards, guidelines, or options in MS rehabilitation [14]—reflecting the absence of any ‘gold standard’ intervention(s) and a need to identify approaches more apt to address the cognitive needs of MS patients [15].

Thus, the problem to be addressed is the lack of treatment options for cognitive difficulties in people with MS. Our planned research will fill the evidence gap through examining the feasibility of trialling a novel online cognitive rehabilitation programme for people with MS. Our approach to cognitive rehabilitation is distinctive from previous interventions (e.g. [16]) in three key respects. Specifically, our approach:

1. Is theory-based, whereas other interventions collate various techniques into a single atheoretical package
2. Employs a focussed, low-intensity cognitive intervention (targeting direct improvement and restoration of cognitive functioning), whereas other interventions include cognitive rehabilitation as part of a broader package (of physical and occupational therapy) complicating future understanding of mechanisms of effect and cost-effectiveness
3. Will train a focal ability, but test for external validity (i.e. whether training transfers to everyday cognition and behaviour), whereas other interventions typically ‘train to test’ (i.e. involve practising final performance assessments, with questionable generalisability beyond this). This latter distinction is important, as meta-analysis has shown that extant cognitive training programmes show weak transferability (i.e. do not generalise beyond train-to-test effects [17]); theoretically, our focal intervention could produce both near- and far-transfer of effects, across indices of cognitive functioning. This was also highlighted as important by our PPI group.

These features also distinguish our (online training) intervention from commercial ‘brain training’ packages (e.g. Lumosity) and other mentally stimulating leisure activities for which the evidence is equivocal at best [18, 19]. Our intervention—*Strengthening Mental Abilities Through Relational Training* (SMART) [20]—is a web-based cognitive training programme that directly trains ‘relational skills’—the skills necessary to understand how concepts relate to one another. SMART is grounded in behavioural science, specifically Relational Frame Theory, which proposes that all human language and complex cognition are underpinned by these relational abilities—such that improving them should enable more rapid and efficient thinking and learning. This proposition from behavioural science is convergent with evidence from education, cognitive science, linguistics, and neuroscience, suggesting that successful cognition involves the ability to relate symbols for functional purposes [21]. Relational skills are developed over time (from infancy) as individuals interact with their environment [22]– and scaffold cognitive abilities such as language, problem-solving, and deductive reasoning [20, 23]. By targeting conceptually and empirically supported core constituents of cognition, SMART can potentially facilitate improved functioning across multiple cognitive domains [20].

In several pilot studies, SMART has shown promise for improving a range of cognitive skills in children [24]. A recent meta-analysis found a moderate effect of SMART on measures of nonverbal intelligence, supporting far-transfer of training, although the primary studies were observed to be at high risk of bias [25]. Whilst most research to date has focussed on increasing scholastic aptitude and general cognitive ability with children, a recent pilot RCT investigated SMART and Treatment-As-Usual (TAU) versus TAU alone (where TAU is pharmacological) for people with Alzheimer’s dementia [26]. Significant small improvements in cognitive abilities were reported for the SMART group at 3-month follow-up. Presti et al. [26] have made the initial steps of transposing SMART into a clinical setting to improve cognitive outcomes for those with deficits and who are in decline, and their results indicate that the programme could be feasibly adapted for use in clinical domains. However, to date, the SMART programme has not undergone rigorous clinical trial evaluation.

In response to the current state of evidence, our work will examine the feasibility of trialling the SMART programme for people with MS. An online programme designed to train relational skills and potentially improve cognitive function may be a cost-effective, accessible intervention for people with MS—addressing unmet patient need for effective cognitive rehabilitation. The theoretical basis of SMART offers advantages over other past-and-ongoing trials and enables us to pursue development in accordance with Medical Research Council (MRC) guidelines for developing complex interventions [27].

### Objective

Primarily, we aim to conduct a feasibility study to inform the development of a definitive trial of SMART for improving cognitive functioning in people with MS. Specifically, we will assess:

1. Acceptability and feasibility of the intervention, delivery format, inclusion/exclusion criteria, baseline and outcome measures, randomisation protocol, and study procedures
2. Participant recruitment and retention rates
3. Sample size needed for fully powered trial

Our secondary objective is to develop the framework for a cost-effectiveness analysis alongside a definitive trial. Our exploratory objective is to assess the signal of efficacy.

## Methods

### Trial design

A three-arm feasibility RCT comparing (1) SMART + treatment-as-usual (TAU) with (2) TAU and (3) active control (‘sham’) training + TAU. We decided on three arms because this will be most informative for the envisaged definitive trial design: It is crucial to include both passive and active control conditions in definitive trials of cognitive rehabilitation, in order to detect any effects over-and-above training-unspecific effects (e.g. [28]. We decided against using a waitlist control because of evidence that waiting-list allocation can have negative effects (e.g. reducing self-management efforts over the waiting period) [29]; moreover, the ethical imperative for providing SMART to all participants is unclear given the as-yet-unknown acceptability and efficacy of this experimental intervention.

### Setting

The study will be set in two hospital-based neurology outpatient clinics for people with MS: in Nottinghamshire, UK (site information available from the corresponding author).

### Eligibility criteria

#### Inclusion criteria

- Diagnosis of MS received ≥3 months pre-enrolment (allowing for acute adjustment, as per other trials of cognitive rehabilitation, e.g. CRAMMS [16])
- Age 18-89 (to meet the standardisation criteria of psychometric assessments)
- Cognitive difficulties as assessed by Perceived Deficits Questionnaire (PDQ) self-report (≥27) and Symbol Digit Modalities Test (SDMT) performance (1.5 SDs or more below the normative reference value)
- Able to read and speak English to the standard necessary for completing assessment and intervention procedures
- Able and willing to access a computer/tablet/smartphone with an internet connection throughout the study
- Able and willing to give informed consent

#### Exclusion criteria

- Currently receiving cognitive rehabilitation
- Previously received SMART training
- Vision or hearing problems precluding completion of procedures

### Interventions

#### TAU

Participants in this arm will receive treatment-as-usual (TAU). Content of TAU for cognitive concerns, based on our clinical experience and knowledge, is often informational support from an MS Nurse with signposting to the MS Society/MS Trust websites.

#### SMART + TAU

Participants in this arm will receive treatment-as-usual (TAU) plus the experimental SMART intervention (theory-based cognitive training).

**Fig. 1:**
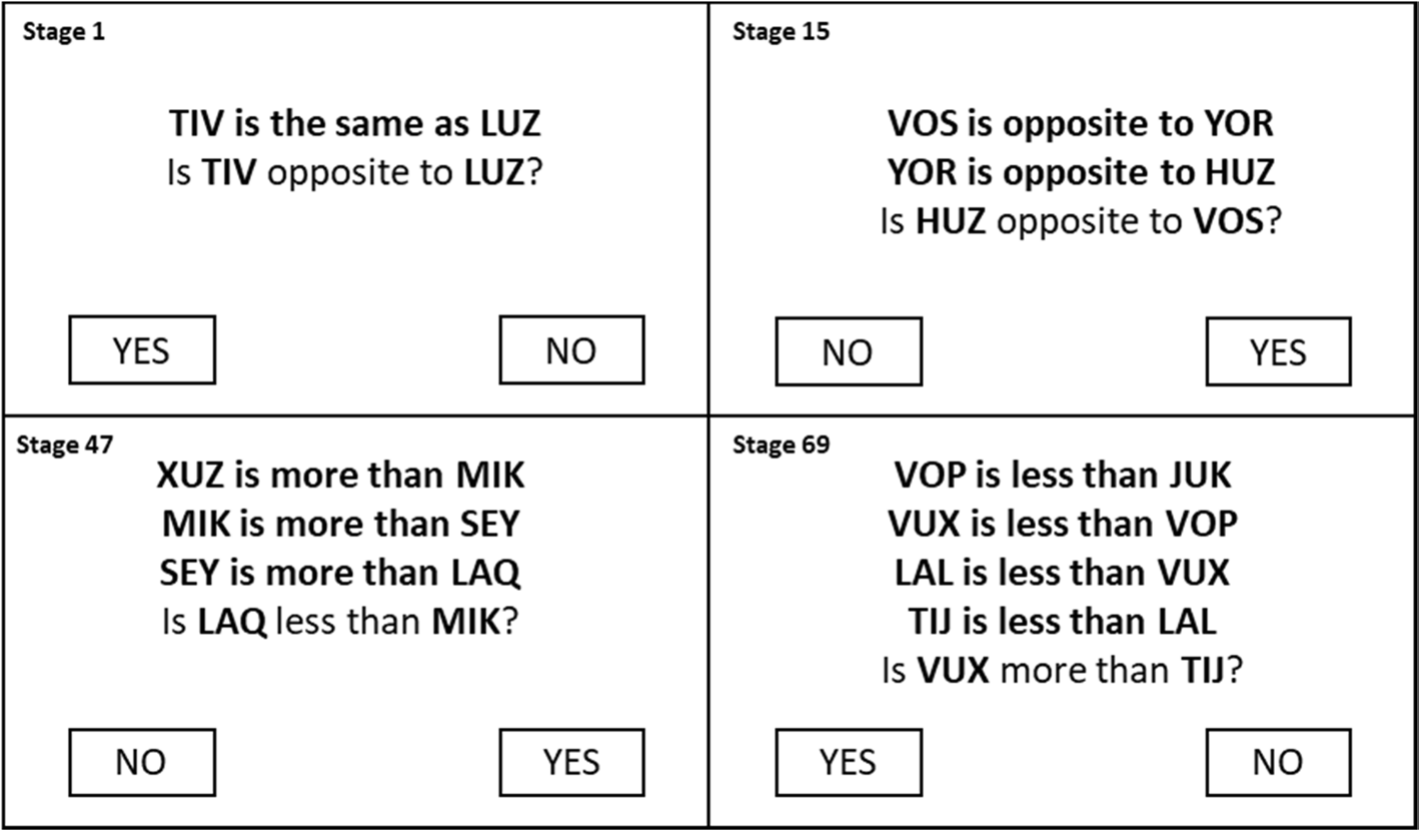
Examples of SMART training tasks of varying complexity

The standard SMART programme will be adapted for intervention [20]—augmenting the standard self-directed programme through facilitator support and the provision of supplementary, accessible guidance materials (based on usability testing with MS patients) [30]. The programme involves presenting a series of logical reasoning problems, with corrective feedback after every response, in the course of training users to derive comparative relationships amongst novel stimuli (‘nonsense words’). The complexity of the problem-solving tasks increases in a stepwise manner over 70 stages of training, requiring increasing relational abilities to progress. Novel stimuli and task configurations are used on every trial, in order to enhance far transfer (i.e. there is no single ‘set’ of correct answers to a single set of specific problem-solving tasks). Figure 1 provides examples of SMART training trials of varying complexity. Please see Additional file 1 for the Template for Intervention Description and Replication (TIDieR) checklist [31] which has been used to describe the intervention to facilitate replication of the intervention in the future, showing the different aspects that are required of an intervention (i.e. who delivers the intervention, how often, when and where).

Participants are typically encouraged to complete the SMART intervention for 30min per session, for a total of 1.5hours per week. However, SMART is incremental and can be completed at the participant’s own pace. Each stage includes a training and a test phase. During training, the participant is required to respond correctly to 16 consecutive exemplar tasks for that stage, within a time limit (typically 30 s per exemplar, to ensure fluency). Tasks continue until this criterion is reached, with corrective audio-visual feedback (‘correct’ or ‘wrong’) provided after each response. Mastery of training is confirmed by performance in a test phase: wherein the learner must respond correctly to a single finite block of 16 consecutive exemplar tasks without feedback. If they pass, they move onto the next stage. If they do not pass, they are directed to repeat both the training and test phase for that stage. It is expected that it would take approximately 12 weeks to complete all 70 stages of the intervention. However, participants will not be required to complete a specific number of stages. Improved cognitive performance has been shown for participants completing just 15 stages (on average) with no clear linear relationship between training completion and outcomes [18].

The intervention will be accessible to participants via their personal computer, tablet, or smartphone with an internet connection. A resource guide prepared for this study will be provided: providing technical information on accessing the programme and a description of how to work through each stage, including a visual chart for progress tracking. Additionally, participants will receive telephone support from an Assistant Psychologist to facilitate intervention access and use. Training activity will be automatically logged for monitoring.

#### Active control (‘sham’) training + TAU

Participants in this arm will receive treatment-as-usual (TAU) plus a control (sham) cognitive training intervention: Sudoku. We selected Sudoku to control for expectancy effects based on popular conceptions that it broadly improves cognitive functions [19, 32], coupled with little evidence supporting this notion [33], and its use as an active control in similar trials [34]. Control training will be delivered online, over the same timeframe/regimen as SMART treatment, and with telephone support to facilitate access—controlling for modality, schedule of engagement, and relational support. To harness expectancy effects [25], we will deliver the control training using a commercial brain training platform—for which there is some published evidence of acceptability and effectiveness in people with MS [35]—where participants will access a study-specific, SMART MS-branded programme. Training activity will be automatically logged.

### Outcomes

#### Primary endpoints

The Primary endpoints in this study relate to the feasibility of proceeding to a Phase III trial. The primary endpoints are based on:

- Acceptability and feasibility of trial procedures
- Appropriateness of eligibility criteria, baseline and outcome measures, audio recording of support sessions, and randomisation protocol
- Recruitment and retention rates
- Intervention acceptability, including progression and completion rates
- Estimating the sample size needed for a Phase III RCT
- Completion rates of outcome measures

#### Secondary endpoints

The secondary endpoints are related to developing a cost-effectiveness framework for a Phase III trial, based on:

- Establishing methods for estimating intervention resource use and costs
- Feasibility of our bespoke service and resource use questionnaire
- Acceptability of the outcome measures for use in estimating the cost-per-QALY of the intervention

#### Exploratory endpoints

The exploratory endpoints are related to the signal of efficacy and indicative estimation of intervention effects (effect sizes and 95% CIs) for the following outcome measures:

Primary outcome measures for exploratory estimation of effects:

- Perceived Deficits Questionnaire (PDQ) | Subjective cognitive functioning
- Repeatable Battery for the Assessment of Neuropsychological Status (RBANS) | Objective cognitive performance (attention, language, visuospatial/constructional abilities, and immediate and delayed memory)
- Symbol Digit Modalities Test (SDMT) | Objective cognitive performance (processing speed)

Secondary outcome measures for exploratory estimation of effects:

- Generalized Anxiety Disorder Scale-7 (GAD-7) | Anxiety
- Patient Health Questionnaire-9 (PHQ-9) | Depression
- Modified Fatigue Impact Scale–5-Item (MFIS-5) | Fatigue
- Personal Questionnaire (PQ) | Participant-identified cognitive problems
- EQ-5D-5L | Health-related quality of life
- MS Impact Scale–29 (MSIS-29) | MS-specific health-related quality of life
- ICECAP-A | Capability wellbeing

### Participant timeline

Table 1 depicts the schedule of enrolment, interventions, and assessments for participants. Over the course of screening and baseline assessment procedures, consenting eligible patients will complete a cognitive assessment battery and questionnaires assessing the impact of living with MS, health-related quality of life, subjective cognitive difficulties, and service/resource use. After completing baseline assessments, participants will be randomly allocated to one of the three intervention arms (using block randomisation with varying block sizes to balance participant numbers across arms). Participants will complete follow-up assessments at 3 and 6 months post-randomisation; after the first follow-up assessment (>3 months post-randomisation) a sub-sample of participants will engage in feasibility-feedback interviews.

**Table 1:** Schedule of enrolment, interventions, and assessments

Procedure/assessment	Screen 1	Screen 2	Baseline (0 months)	*Intervention period*	FU1 (3 months)	Interval b/w FUs	FU2 (6 months)
Enrolment:							
Initial eligibility screen	X						
Informed consent	X						
Demographic information	X						
Randomisation			X				
Interventions:							
SMART + TAU				X			
Active control training + TAU				X			
TAU				X			
Assessments:							
PDQ	X				X		X
Symbol Digit Modalities Test (SDMT)		X			X		X
RBANS			X		X		X
Generalized Anxiety Disorder Scale-7 (GAD-7)							
Patient Health Questionnaire-9 (PHQ-9)			X		X		X
Modified Fatigue Impact Scale–5-Item (MFIS-5)			X		X		X
Personal Questionnaire (PQ)			X		X		X
EQ-5D-5L			X		X		X
MS Impact Scale–29 (MSIS-8D; v.2)			X		X		X
ICECAP-A			X		X		X
Service- and resource-use questionnaire			X		X		X
Feedback interviews						X^a^	

*FU* follow-up, *Screen* screening appointment, *ICECAP-A* Investigating Choice Experiments Capability Measure for Adults, *TAU* treatment-as-usual

^a^With selected participants who will be consented prior to the interview

### Sample size

For the purposes of the current study, we will continue to approach and recruit people until we have randomised 60 participants (20 participants per arm). This should provide us with sufficient information in informing the design of a Phase III RCT. Twelve participants per arm would serve to inform trial feasibility outcomes and provide minimally sufficient precision for preliminary parameter estimates [36, 37]. We will recruit 20 per arm because (1) with 20 per arm, our 95% confidence intervals for key estimates (such as trial retention and intervention completion rates) will be narrow enough (<±20%) that, in terms of our criteria for progression to a definitive trial, there will be no substantive misclassification (‘red’ as ‘green’ or vice-versa; please see Table 2) and (2) at least 15 per arm is recommended for estimating variance to inform sample size requirements for a 90% powered main trial aiming to detect effects of moderate (clinically meaningful) magnitude [38]. Given service-activity data and prevalence of cognitive difficulties in people with MS, this target is achievable over a 12-month recruitment phase.

**Table 2:** Feasibility assessment

Question	Data	Analysis	Progression criteria
Feasibility of the study	Interviews (i.e. post-trial feasibility interviews)—e.g. acceptability of randomisation; recommended changes to procedures	Framework analysis (structured, purposive coding of qualitative content)	Formative (included to support design of a definitive trial, but no assigned progression criteria cut-offs)
Acceptability of intervention	Drop-out rate (and reasons for withdrawal)	Frequencies/percentages	≥80% of SMART group complete ≥6 sessions (green), 79-60% complete ≥6 sessions (amber), <60% complete ≥6 sessions (red)
	Intervention progression and completion rates	Descriptive statistics/average percentage completion (and range)	Formative
	Interviews	Framework analysis	Formative
*Credibility* of intervention	Interviews (plus relevant comments within session recordings)	Framework analysis	Formative
Feasibility of measures	Missing response-data	Percentage	Formative
	Estimates of completion time	Descriptive statistics	Formative
	Late completion	Percentage	Formative
	Interviews	Framework analysis	Formative
Feasibility of framework for cost-effectiveness analysis	Missing data on intervention resource use	Percentage	Formative
	Missing response data—QALY measures and service and resource use questionnaire	Percentage	Formative
	Estimates of time taken to complete measures - QALY measures and service and resource use questionnaire	Descriptive statistics	Formative
Feasibility of framework for cost-effectiveness analysis (cont…)	Late completion of measures—QALY measures and service and resource use questionnaire	Percentage	Formative
Feasibility of recruitment and randomisation	Numbers of outpatients eligible	Frequencies/percentages	Formative
	Numbers eligible who expressed interest/discussed w/researcher	Frequencies/percentages	Formative
	Numbers consenting/randomised	Frequencies/percentages	≥80% of planned (green), 79–60% of planned (amber), <60% of planned (red)
	Reasons for non-participation? (e.g. online modality as barrier? Unwilling to be randomised?)	Frequencies/percentages	Formative
	Interviews	Framework analysis	Formative
Feasibility of retention within the study	Number completing baseline and outcome assessments at Follow-up 1 (3 months)	Frequencies/percentages	≥80% completing to follow-up 1 (green), 79–-60% completing (amber), <60% completing (red)
Signal of efficacy	Indicative estimation of intervention effects	Estimates of (group-level) effect sizes with 95% CIs and proportions achieving reliable/clinically significant change	Red: 95% CIs exclude the possibility of a meaningful effect size for our screening measures (based on reliable-change criteria)

### Recruitment

Participants will be recruited from MS clinics in two centres (Nottingham City and North Nottinghamshire). Recruitment will first be opened in the Nottingham site and, depending on the recruitment rates, we will secondarily open recruitment in the North Nottinghamshire site. The initial approach will be from a member of the patient’s usual care team (MS Nurses or Neurologists), and information about the trial will be on display in the relevant clinical areas.

#### Postal invitation

In the recruiting MS clinics, the clinical teams have regular contact with all people diagnosed with MS in the community. The clinical staff will identify potential participants from hospital records. An invitation letter and Participant Information Sheet (PIS) will be sent to identified patients via post or email by the MS Nurses or a member of the clinical team. This invitation letter will include study information and research team details. Patients who are interested will contact the research team—whereupon a researcher will address any questions, ensure their understanding of the study, and arrange screening procedures. The researcher will explain that the screening procedures are to check that the patient meets the study inclusion criteria.

#### Face-to-face invitation

In addition to the invitation letter, potential participants who attend clinic visits can be introduced to the study by their neurologist or MS nurses and given/sent the PIS. People who do not contact the research team will have a single phone call by the clinical team to enquire whether they remember receiving the PIS and whether they would like further information about the study, where possible. If they do not wish to have further information, no further contact will be made by the researchers. If, however, they wish to have more information, the clinical team will request verbal consent to pass on their contact details to a researcher, who can provide them with more information about the trial. The clinical team will record the date and time when verbal consent was obtained to pass on contact details. The research team will then contact potential participants to address any questions, ensure their understanding of the study, and enquire whether they are still interested in taking part. If so, screening procedures will be arranged—the researcher will explain that the screening procedures are to check that the patient meets inclusion criteria.

Screening appointments will be arranged as suitable to the potential participant: The first screening procedure (completion of PDQ and demographic information) can be undertaken via an online survey platform or via telephone/video call with a researcher; the second screening procedure (completion of RBANS) must be undertaken with a researcher either via video call or in-person. Potential participants will be sent a Consent Form (CF) and (if requested) another copy of the PIS in advance of the first screening appointment so that they have sufficient time and information to understand the study before consenting to the study and engaging in screening procedures.

We will explain to the potential participant that entry into the trial is entirely voluntary and that their treatment and care will not be affected by their decision, and that they can withdraw at any time. In the event of their withdrawal, it will be explained that their data collected so far may not be erased in accordance with the University’s Research Privacy Notice and information given in the Participant Information Sheet and we will seek consent to use the data in the final analyses where appropriate. If participants withdraw from the study interventions, the study team will ask if they are willing to remain in the trial and complete trial assessments. As this is a feasibility study, recruitment will continue until at least 60 participants have been randomised (20 to each group).

Participants will not be paid to participate in the trial. Travel expenses will be offered for any visits in excess of usual care.

### Randomisation and blinding

Participants will be individually randomised at baseline (after consent) in equal proportions to one of three groups (1:1:1 ratio) using block randomisation in permuted blocks of three and six.

Treatment allocation will be computer generated via the electronic trial database in Castor EDC (Castor Electronic Data Capture, available at: https://castoredc.com). Allocation sequence is concealed by Castor EDC until the randomisation of a participant. Castor EDC is used to assign participants to different groups.

Given the nature of the intervention, participants and the intervention-facilitating researchers will not be blinded. No unblinding procedures relating to potential adverse effects are therefore required. The assessments will be conducted by a Research Fellow who will be blind to treatment allocation. We will record any instances of unblinding to assess the feasibility of blinding outcome assessors to allocation.

### Data collection methods

#### Screening and baseline measures

PDQ [39]: to assess self-reported cognition (for screening and focal outcome measurement). Perceived cognitive function, measured using the 20-item PDQ, assesses cognitive functions most affected in MS: attention, memory, planning, and organisation. The PDQ is associated with objective cognitive performance in MS [40] and has shown excellent internal consistency (*ɑ* = .93) [39]. We will use a cut-off of ≥27 [41] to identify study eligibility (i.e. 1.5 SDs or more above the normative mean).

*Symbol Digit Modalities Test (SDMT)* [42]: to assess objective cognitive performance (processing speed; for screening and focal outcome measurement). The SDMT [42] is a symbol substitution test that examines processing speed and attention and is reported as the most sensitive test for MS cognitive problems [43]. The SDMT has shown excellent test-retest reliability (*r* = .97) [43]. Age-, education-, and gender-adjusted norms are available [44], and these will be used to define cognitive impairment (for study eligibility) as scoring 1.5 SDs or more below the normative reference value.

The RBANS [45]: to further assess objective cognitive performance (attention, language, visuospatial/constructional abilities, and immediate and delayed memory; for screening and focal outcome measurement). The RBANS is a brief test of cognitive abilities across multiple domains, with domain composite scores derived from 12 subtests (list learning, story memory, figure copy, line orientation, picture naming, semantic fluency, digit span, coding, list recall, list recognition, story recall, figure recall). Subtests also support the derivation of an executive errors scale (reflecting executive functioning) [46]. Normative data provide age- and education-corrected scores [47] and these can be used to further define cognitive impairment as scoring ≥1 SD below the mean on ≥1 RBANS- composite [48]. The RBANS is validated in this population and has various strengths (including alternate forms to enable repeated assessment) [49]; adequate test-retest reliabilities have been found across subtests (with *r* = .80 for the Total Scale) [50].

*Generalized Anxiety Disorder Scale-7 (GAD-*7) [51] *and Patient Health Questionnaire-9* (PHQ-9) [52] to assess distress—an important correlate of cognitive concerns. The GAD-7 and PHQ-9 have been shown to retain construct validity and acceptable internal consistencies for use in MS (*ɑ*s = .75 and .82, respectively) [53, 54]. In this population, scores ≥10 in either anxiety or depression indicate clinical distress [55]

*Modified Fatigue Impact Scale–5-Item (MFIS*-5) [56]: to assess fatigue and its perceived impact on cognitive, physical, and psychosocial functioning. The MFIS-5 has demonstrated excellent internal consistency (*ɑ* = .90) and construct validity [57] and will be applied to identify possible fatiguing effects of the intervention.

*Personal Questionnaire* (PQ) [58]: to assess patient-described cognitive problems and their everyday impact (patient-generated outcome measure). The PQ has demonstrated good internal consistency (*ɑ* = .80) and treatment sensitivity [58].

EQ-5D-5L [59, 60]: provides health state utility values (HSUVs) and is NICE recommended for estimating the cost-per-QALY of interventions [61] (informing cost-effectiveness framework). The EQ-5D-5L has demonstrated good test-retest reliability (ICC = .80) and construct validity in people with MS [62].

*MS Impact Scale–29* (MSIS-8D; v.2): MS-specific QoL to additionally provide MS-specific HSUVs [63, 64] (informing cost-effectiveness framework); this scale has shown good internal consistency (*ɑ*s = .80) and treatment sensitivity [65].

ICECAP-A [66]: Provides capability wellbeing values (reflecting the ability to ‘do’ and ‘be’ the things that are important in life) and is NICE recommended for use in economic evaluations, alongside health measures [67] (informing cost-effectiveness framework). The ICECAP-A has been shown to have acceptable internal consistency (*ɑ* = .74) and construct validity [68].

*Service- and resource-use questionnaire* developed from our previous pilot work (informing cost-effectiveness framework). The questionnaire design has been informed by the Database of Instruments for Resource Use Measurement (DIRUM) [69] and the core resource-use items [70].

These measures were selected because they have adequate psychometric properties, have been used in other trials with this population, are brief, and capture the outcomes our PPI group felt were most important for people with MS.

#### Follow-up measures

All participants will be assessed 3 and 6 months post-randomisation, using the same measures as at baseline. The assessing Research Fellow will be blind to allocation (any instances of unblinding will be recorded).

#### Intervention resource requirements

The resources needed to deliver the intervention (e.g. providing the SMART programme online for people with MS, facilitators’ time) will be assessed via participant case records and discussion with the intervention developers.

#### Feasibility interviews

After the first follow-up assessment, 30 participants (10 from each arm) will be invited to participate in a semi-structured feedback interview. We considered the timing of this interview (whether to wait until after the final follow-up assessment) and ultimately decided that (at least for this feasibility study) we should keep interviews proximal to the main study procedures and intervention—particularly given the cognitive difficulties experienced by our participants and likelihood that later interviews would strain retrospective recall. Table 1 outlines the overall schedule of study assessments/procedures (including how the feedback interviews fit within this). We will use ‘maximum variation’ sampling to select a demographically and clinically diverse sample. We anticipate theoretical sufficiency with 30 participants but will extend recruitment if needed. We will use the Theoretical Framework of Acceptability to guide our interviews and analyses [71]. These interviews will allow SMART participants to feed-back on what they found helpful/unhelpful about intervention content and delivery, enabling us to further refine these. For those in control arms, interviews will explore their feelings about not receiving the intervention. All participants will be asked about the acceptability of research processes (including randomisation).

Participant feedback is often subject to the ‘halo effect’ produced by a perceived lack of independence of assessors. We will therefore train and supervise two patient-partners (PPs) to help us in this process, with support from the Research Fellow who is independent to intervention delivery. The process of involving PPs in conducting interviews engenders agency and capacity-building and enables PPs to bring their unique perspectives of being fellow patients, which may permit research-participants to be more open. We successfully used such PP engagement in the CRAMMS trial [16].

Up to 10 feedback interviews will be conducted by our PPs, who will be trained and supported to conduct them (by our PPI Lead), and the remainder will be conducted by the Research Fellow. PPs will have had DBS checks and will only conduct telephone/video-call interviews, following our Trust’s Volunteer Policy.

An interview schedule will be developed/piloted with the PPI group and PPs. Interviews will be audio-recorded with permission.

### Data management

All study staff and investigators will comply with the principles of the Data Protection Act (2018) in protecting the rights of study participants with regard to the collection, storage, processing, and disclosure of personal information and will uphold the Act’s/Regulations core principles. Each participant will be assigned a study identity number, for use on CRFs other trial documents and the electronic database. Personal data, research data and the linking code will be stored electronically in separate locations: this will include using encrypted digital files within password-protected folders and storage media. Personal information shall be stored separately to research data and will be kept secure and maintained. Personal data will be stored for 6 months following the end of the study, so that the Chief Investigator may provide participants with a summary of the research (should they wish to receive a copy). Data generated through this study will be available for inspection on request by the participating physicians, the University of Lincoln representatives, the REC, local R&D Departments and the regulatory authorities. Routine reviews of submitted data will be conducted to identify and follow up on missing data, inconsistent data, data outliers, and potential protocol deviations that may be indicative of systematic or significant errors in data collection and reporting at a site.

Questionnaire and eCRF data will be collected and stored using Castor EDC. Data gathered using the Castor EDC platform will only be accessible to the research team, with access rights managed by the database manager (ER). Participants will be given a unique study identifying code so that no identifiable information need to be entered.

### Data-Analysis

To specifically address the feasibility objectives of the proposed programme, our analysis will draw on multiple data sources—including qualitative data from post-trial feasibility interviews (see Table 1). Quantitative analyses (conducted using R and SPSS) will be primarily descriptive, focussing on key indicators of trial and cost-effectiveness analysis viability—including recruitment/attrition rates. Variability estimates will be computed for study outcomes and used to inform sample-size calculations for the definitive RCT (following DELTA [2] guidance [72]). To identify the signal of efficacy, we will estimate (group-level) effect sizes (with 95% CIs) and proportions achieving (individual-level) reliable and clinically significant changes. To handle incomplete outcome data when testing for the signal of efficacy (whether confidence intervals around effect sizes preclude clinically important differences) we will estimate effects using intent-to-treat linear mixed modelling—an available-case method that can accommodate missing datapoints. A Statistical Analysis Plan has been developed by the trial statistician, in consultation with the UoL Clinical Trials Unit, and will be applied with oversight from the TSC/DMEC. Qualitative data will be purposively analysed—applying Framework Analysis [73, 74] with support of Nvivo software—to understand participant study experiences and identify areas for development/revision towards a definitive trial. Framework Analysis is a structured analytical approach, enabling us to rapidly appraise data in relation to our a priori feasibility questions and deductive application of the Theoretical Framework of Acceptability [71].

### Monitoring

Study conduct will be governed by a Joint Trial Steering Committee and Data Monitoring (and Ethics) Committee (TSC/DMEC)

#### Joint TSC/DMEC

The TSC/DMEC will have an independent chair, two clinical/academic members, two Patient and Public Involvement (PPI) members, and an independent statistician. The TSC/DMEC will provide independent oversight of the study and will meet (in person or by teleconference) at least every 6 months with more frequent meetings as necessary. This joint committee will safeguard the interests of trial participants—with particular reference to safety and the efficacy of the intervention—monitor the overall progress and conduct of the trial, monitor the outcome data regularly during data collection, and assist and advise the investigators so as to protect the validity and credibility of the trial.

#### Harms

Adverse events of participation in this study may be (1) exacerbation of MS-related fatigue through engagement with the intervention and study procedures and (2) elevated distress if participants find that they are not performing as well as they think they should during cognitive assessments. There are no serious adverse events anticipated with participating in this study. In practice, with respect to (1), participants will be able to withdraw at any point—one purpose of this (feasibility) study is to understand whether the intervention and study procedures are acceptable, and withdrawal due to perceived burdensomeness/exacerbation of fatigue will be informative for addressing our feasibility aims and informing future intervention and trial design. With respect to (2), this is considered to be a low-probability risk, causing minimal distress (based on our experiences of running similar trials (e.g. CRAMMS [10.1186/ISRCTN09697576]) but any such distress will be managed by the assessing psychologists who are qualified to deal with distress in an appropriately compassionate manner—and will make necessary referrals (to the participant’s GP) as needed.

All adverse events will be recorded and closely monitored until resolution, stabilisation, or until it has been shown that the study treatment/intervention is not the cause.

Participant removal from the study due to adverse events. Any participant who experiences an adverse event may be withdrawn from the study at the discretion of the Investigator.

### Auditing

Compliance with the protocol will be assessed throughout using central monitoring techniques. This will be achieved through routine reviews of submitted data to identify and follow-up on missing data, inconsistent data, data outliers, and potential protocol deviations that may be indicative of systematic or significant errors in data collection and reporting at a site. An interim analysis will be conducted by the TSC/DMEC to check that the project is operating appropriately—examining consent, retention, and completeness of data. The TSC/DMEC can make a recommendation for continuation/stopping or highlight any concerns or areas that may need attention (e.g. changes in recruitment practices, strategies to improve retention, or similar).

Accidental protocol deviations may occur at any time. Accidental protocol deviations will be adequately documented on the relevant forms and reported to the Chief Investigator and Sponsor immediately. Deviations from the protocol which are found to frequently recur are not acceptable, these will require immediate action and could potentially be classified as a serious breach.

### Dissemination policy

Dissemination will be multipronged to inform a wide audience of patients, carers, and clinicians.

1. Trial participants will be offered a lay summary of findings.
2. The wider public will be informed through Trust- and study-specific websites, and via press offices for the collaborating institutions—care will be taken to reflect the early staging of the research, focus on feasibility, and conditionality of potential implications.
3. We will submit findings for presentations at relevant meetings: informing the academic community and fostering interest from potential collaborators for the definitive RCT (extending the network of research sites).
4. We will publish feasibility results in (open access) peer-reviewed national and international journals and professional newsletters.
5. Along with our PPI Advisory Panel, we will co-write for newsletters/webpages of relevant charities that reach patients and carers directly.

Regarding outcomes, study results will directly inform protocol development for a fully powered, definitive RCT. Should this RCT demonstrate that SMART is clinically effective, there is a clear trajectory to benefit for patients, carers, and the NHS: increasing the availability and accessibility of treatment/self-management options for cognitive rehabilitation in MS—and thereby enabling improved service provision and reducing demands on services to manage sequelae of untreated cognitive deficits. If clinically effective, the low-resource nature of the intervention makes SMART more likely to be implemented—and a full cost-effectiveness analysis in the future RCT will indicate the relative value for money to the NHS/Personal Social Services of SMART. The remote accessibility of SMART (as an online intervention) is particularly beneficial in the context of COVID-19 and would enable swift and scalable implementation—consistent with the NHS digital healthcare agenda.

### Protocol version and amendments

This publication is based on protocol version 2.0_25.11.2021.

### Sponsor information

Sponsorship for this study is provided by the University of Lincoln (Sponsor ID 21002).

## Discussion

Regarding outcomes, study results will directly inform protocol development for a fully powered, definitive RCT. Should this RCT demonstrate that SMART is clinically effective, there is a clear trajectory to benefit for patients, carers, and the NHS: increasing the availability and accessibility of treatment/self-management options for cognitive rehabilitation in MS—and thereby enabling improved service provision and reducing demands on services to manage sequelae of untreated cognitive deficits. If clinically effective, the low-resource nature of the intervention makes SMART more likely to be implemented—and a full cost-effectiveness analysis in the future RCT will indicate the relative value for money to the NHS/Personal Social Services of SMART. The remote accessibility of SMART (as an online intervention) is particularly beneficial in the context of COVID-19 and would enable swift and scalable implementation—consistent with the NHS digital healthcare agenda.

## Supplementary Information

**Additional file 1.** Template for intervention description and replication (TIDieR) checklist for the SMART MS intervention.

## Data Availability

This is the study protocol of the in-progress study and data are not yet available. Anonymised quantitative participant data will be made available via an appropriate publicly available repository service. Qualitative participant data (such as interview transcripts) will not be made publicly available, as this could compromise participant anonymity. Data will be summarised on clinicaltrials.gov. As a requirement of our grant contract, the outputs of this study will be made available via researchfish.

## Abbreviations

AE: Adverse event
CF: Consent form
CI: Chief Investigator
CRF: Case Report Form
CTU: Clinical Trials Unit
DMEC: Data Monitoring (and Ethics) Committee
GCP: Good Clinical Practice
HRA: Health Research Authority
HTA: Human Tissue Authority
ICF: Informed consent form
ISF: Investigator Site File (This forms part of the TMF)
ISRCTN: International Standard Randomised Controlled Trials Number
LIH: Lincoln Institute for Health
LinCTU: Lincoln Clinical Trials Unit
MS: Multiple sclerosis
NHS R&D: National Health Service Research & Development
PI: Principal Investigator
PIC: Participant Identification Centre
PIS: Participant Information Sheet
PPI: Patient and Public Involvement
RCT: Randomised control trial
REC: Research Ethics Committee
SAE: Serious adverse event
SOP: Standard operating procedure
TMF: Trial Master File
TMG: Trial Management Group
TSC: Trial Steering Committee
UoL: University of Lincoln
