# Supplementary material for: Strengthening Mental Abilities with Relational Training (SMART) in multiple sclerosis (MS): study protocol for a feasibility randomised controlled trial
Source: Pilot Feasibility Stud. 2022 Sep 3;8:195. doi: 10.1186/s40814-022-01152-7 (PMC9439942; doi:10.1186/s40814-022-01152-7)
Supplement: Supplementary file 1 — Additional file 1. Template for intervention description and replication (TIDieR) checklist for the SMART MS intervention. [file 40814_2022_1152_MOESM1_ESM.docx]

**Additional file 1**

Below we use the template for intervention description and replication (TIDieR) checklist^(1)^ to provide a structured description of the study intervention:

| 1.   What is the **NAME** of the intervention? | Strengthening Mental Abilities through Relational Training (SMART) |
| --- | --- |
| 2.   **WHY** do the intervention? | *Intervention rationale:*  Cognitive difficulties are common amongst people with MS, with up to 70% experiencing deficits in higher-level brain functions – such as planning and problem-solving, attention, and memory. In a national survey, these cognitive difficulties were identified as the most debilitating and distressing consequence of MS^(2)^.  Cognitive rehabilitation is not routinely offered in the NHS^(3)^ – and, when it is offered, largely focuses on teaching people to compensate for deficits (e.g., using external memory aids) rather than retraining cognitive skills. A recent state-of-the-field review from international MS experts has called for research towards identifying effective, evidence-based, and clinically feasible interventions to address cognitive deficits in MS^(4)^ . There is evident uncertainty about how to intervene effectively, and this is recognised by the James Lind Alliance as a top-10 priority area for research-funding^(5)^.  As evidenced in reviews by our team and others^(6-8)^, the efficacy of cognitive rehabilitation for people with MS remains weak and inconclusive.  To date, studies of cognitive rehabilitation in MS have not been predicated on a clear theoretical rationale for intervention, making it difficult to synthesise evidence and understand or optimise effects. SMART is distinct from other interventions in that it is theory-based; employs a focussed, low-intensity cognitive intervention (targeting direct improvement and restoration of cognitive functioning); and theoretically enables both near- and far-transfer of effects, across indices of cognitive functioning. |
|  | *What are the underpinning theories?*   - SMART^(9)^ is grounded in behavioural science, specifically Relational Frame Theory, which proposes that all human language and complex cognition is underpinned by relational abilities – abilities necessary to understand how concepts relate to one another – such that improving these should enable more rapid and efficient learning, clearer thinking, and fluency in making sense of new information. - This proposition from behavioural science is convergent with evidence from education, cognitive science, linguistics, and neuroscience, suggesting that successful cognition involves the ability to relate symbols for functional purposes^(10)^. Relational abilities are developed over time (from infancy) as individuals interact with their environment^(11)^ – and scaffold cognitive skills such as language, problem solving, and deductive reasoning^(9, 12)^. - By targeting conceptually and empirically supported core constituents of cognition, SMART can potentially facilitate improved functioning across cognitive domains^(9)^. - PPI views and feedback (Focus groups with people with MS and their carers/families):   - Need for treatments that can improve cognitive functioning   - Need for treatments that are easy to access from home   - Important that treatments have broad effects with everyday impact (not just ‘training to test’)   - SMART appears acceptable *in principle* and should be tested with (and tailored for) people with MS   - Motivated to try SMART |
| 3.   **WHAT** **materials** are needed for the intervention? | *Provided intervention materials (refined through pre-trial testing)*   - Access to online MS-adapted SMART programme for cognitive rehabilitation - Guide to accessing the SMART programme (document) |
|  | *Facilitator materials (developed through pre-trial testing):*   - Assistant Psychologist manual for facilitating patient intervention access and use (document) |
|  | *Extant participants materials:*   - Personal computer, tablet, or smartphone with internet connection |
| 4.   **WHAT Procedures** take place in the intervention? | *Provider training:*   - Training for facilitator (Assistant Psychologist) from Research Fellow experienced in using SMART with advisory input from Co-Investigator Bryan Roche (as developer of SMART and underpinning theory) - Ongoing supervision for facilitator (provided by CI with support from other experienced clinical psychologists in the team) |
|  | *Intervention:*  SMART in an online cognitive training programme that directly trains relational abilities (which scaffold broader cognitive skills, such as learning, reasoning, and problem-solving). Patients access from home with an informational resource and facilitator telephone-support to enable access.  The programme involves a series of logic problems that train participants to derive comparative relationships among novel stimuli – which are arbitrary nonsense syllables based on English language cues. Difficulty increases over 70 stages of training, requiring increasing relational abilities to progress. Each stage includes a training and a test phase. During training, the learner is required to respond correctly to 16 consecutive exemplars of that stage, within a time limit (typically 30 seconds per exemplar). Tasks continue until this criterion is reached. Audio-visual feedback (‘correct’ or ‘wrong’) is provided. In testing, the learner must respond correctly to a single ﬁnite block of 16 consecutive exemplars without feedback. If they pass, they move onto the next stage. If they do not pass, they are directed to repeat both the training and test phases for that stage. |
| 5.   **WHO** provides the intervention? | Facilitator support will be provided by an Assistant Psychologist who is trained and supervised in supporting the intervention |
| 6.   **HOW** is the intervention delivered? | Provided individually, online (via a web browser) with telephone support from facilitator (adapted to patient need and preference) |
| 7.   **WHERE** is the intervention delivered? | Accessible remotely from home (via internet-connected computer/tablet/smartphone) using individually allocated login details. |
| 8.   **WHEN** is the intervention delivered? **HOW MUCH**? | Participants are typically encouraged to complete the SMART intervention for 30min per session, for a total of 1.5hours per week. However, SMART is incremental and can be completed at the participant’s own pace.  It is expected that it would take approximately 12 weeks to complete all 70 stages of the intervention. However, participants will not be required to complete a specific number of stages. |
| 9.   How is the intervention **TAILORED**? | *Tailoring occurs through:*   - Flexible access and pacing (SMART can be accessed as/when/where convenient, at the participant’s own pace) - Incremental staging, feedback, and progression criteria (complexity only increases with mastery) - Delivery of facilitation support (based on individual needs and preferred schedule) - Assessment and monitoring of patient-described problems (patient-generated outcome measure [Personal Questionnaire]) |
| 10. **MODIFICATIONS** to the intervention | Based on pre-trial piloting and cognitive interviews, we have adapted the standard SMART programme to maximise suitability for people with MS: Providing additional instructional materials and individually tailored facilitator support |
| 11. **HOW WELL Planned?** (How the intervention adherence and fidelity will be assessed?) | *How and by whom:*  We will request facilitator and patient consent to audio-record sessions. When this is not possible, the facilitator will keep detailed notes.  We will have full logs of participant intervention-use (including individual-level data on frequency and duration of access, and training performance and progression) via the online SMART platform. |
|  | *Strategies used to maintain/improve fidelity:*  These recorded sessions will be compared to the facilitator guide materials. We will map data onto the key elements of content, coverage, frequency, and duration – attending to facilitation strategies, quality of delivery, and participant responsiveness^(13)^ |
| 12. **HOW WELL Actual?** (the extent to which the intervention was delivered as planned) | *This will form a focus of the feasibility trial* |

**References**

1. Hoffmann TC, Glasziou PP, Boutron I, Milne R, Perera R, Moher D, et al. Better reporting of interventions: template for intervention description and replication (TIDieR) checklist and guide. Bmj. 2014;348.

2. Dorning H, Luck G, Holloway E. A lottery of treatment and care - MS services across the UK. MS Society; 2013.

3. Klein OA, Das Nair R, Ablewhite J, Drummond A. Assessment and management of cognitive problems in people with multiple sclerosis: A National Survey of Clinical Practice. International journal of clinical practice. 2019;73(3):e13300.

4. Sumowski JF, Benedict R, Enzinger C, Filippi M, Geurts JJ, Hamalainen P, et al. Cognition in multiple sclerosis: State of the field and priorities for the future. Neurology. 2018;90(6):278-88.

5. James Lind Alliance. Multiple Sclerosis Top 10 priorities 2019 [Available from: http://www.jla.nihr.ac.uk/priority-setting-partnerships/multiple-sclerosis/top-10-priorities/.

6. das Nair R, Martin KJ, Lincoln NB. Memory rehabilitation for people with multiple sclerosis. Cochrane Database of Systematic Reviews. 2016(3).

7. Mitolo M, Venneri A, Wilkinson ID, Sharrack B. Cognitive rehabilitation in multiple sclerosis: a systematic review. Journal of the Neurological Sciences. 2015;354(1-2):1-9.

8. Rosti‐Otajärvi EM, Hämäläinen PI. Neuropsychological rehabilitation for multiple sclerosis. Cochrane Database of Systematic Reviews. 2014(2).

9. Cassidy S, Roche B, Colbert D, Stewart I, Grey IM. A relational frame skills training intervention to increase general intelligence and scholastic aptitude. Learning and Individual Differences. 2016;47:222-35.

10. McLoughlin S, Tyndall I, Pereira A. Relational Operant Skills Training Increases Standardized Matrices Scores in Adolescents: A Stratified Active-Controlled Trial. Journal of Behavioral Education. 2020:1-28.

11. Hayes SC, Barnes-Holmes D, Roche B. Relational frame theory: A post-Skinnerian account of human language and cognition: Springer Science & Business Media; 2001.

12. Cassidy S, Roche B, Hayes SC. A relational frame training intervention to raise intelligence quotients: A pilot study. The Psychological Record. 2011;61(2):173-98.

13. Carroll C, Patterson M, Wood S, Booth A, Rick J, Balain S. A conceptual framework for implementation fidelity. Implementation science. 2007;2(1):40.
